# A Genome-Wide Association Study Links *AEBP2* to Litter Size in Meishan Pigs

**DOI:** 10.3390/ani16203198

**Published:** 2026-10-12

**Authors:** Mengqian Cao, Caifeng Wu, Weilong Tu, Yeqing Gan, Yi Bian, Xin Gu, Wengang Zhang, Shushan Zhang, Lingwei Sun, Jiehuan Xu, Mengqian He, Defu Zhang, Jun Gao, Jianjun Dai

**Affiliations:** 1 Institute of Animal Science and Veterinary Medicine, Shanghai Academy of Agricultural Sciences, Shanghai 201106, China; 13887223118@163.com (M.C.); 20150646@saas.sh.cn (C.W.); tuweilong@saas.sh.cn (W.T.); zhangshushan@saas.sh.cn (S.Z.); sunlingwei@saas.sh.cn (L.S.); jiehuanxu@saas.sh.cn (J.X.); hemengqian@saas.sh.cn (M.H.); zhangdefu@saas.sh.cn (D.Z.); 2 Shanghai Municipal Key Laboratory of Agri-Genetics and Breeding, Shanghai 201106, China; 3 Key Laboratory of Livestock and Poultry Resources Evaluation and Utilization, Ministry of Agriculture and Rural Affairs, Shanghai 201106, China; 4 Shanghai Engineering Research Center of Breeding Pig, Shanghai 201106, China; 5 Shanghai Meishan Pig Breeding Centre, Jiading District, Shanghai 201807, China; 18930381338@163.com (Y.G.); 18021069959@163.com (Y.B.); 6 Shanghai Animal Disease Prevention and Control Center, Shanghai 200050, China; guxun69@gmail.com (X.G.); geligaori@163.com (W.Z.)

**Keywords:** Meishan pig, litter size, genome-wide association analysis, *AEBP2*, porcine ovarian granulosa cells

## Abstract

This study combined liquid-phase chip technology with a genome-wide association study (GWAS) to identify candidate functional genes for litter size in Meishan pigs. Genotyping of 198 Meishan sows using the “Shenxin No. 1” 60K porcine SNP chip, followed by quality control with PLINK v1.90, yielded 31,430 autosomal SNPs. Principal component analysis revealed no significant population stratification. Using a mixed linear model in GEMMA (v 0.98.1), we identified 9 significant SNP loci and 52 candidate genes on chromosomes 5 and 14, including *AEBP2*, *P2RX2*, *ANKLE2*, *PGAM5*, and *EP400*, which are implicated in embryonic development, ovarian function, and cell proliferation. Isolated porcine ovarian granulosa cells were validated by FSHR immunofluorescence, showing 99% purity. Overexpression of *AEBP2* significantly upregulated its protein expression (Western blot) and promoted granulosa cell proliferation (CCK-8). Collectively, these results pinpoint potential candidate genomic regions and functional genes associated with litter size and suggest that *AEBP2* may enhance reproductive performance by promoting granulosa cell proliferation and follicular development. This provides new targets for elucidating the genetic mechanisms underlying the high fecundity of Meishan pigs and for advancing molecular breeding efforts.

## Simple Summary

This study searched for genes that affect the number of piglets born in Chinese Meishan pigs, a breed famous for having large litters. By scanning the pigs’ genomes and testing one particular gene, *AEBP2*, we found that it can stimulate the growth of ovarian cells that are crucial for egg maturation. These findings may guide future efforts to breed pigs with even better reproductive performance.

## 1. Introduction

As a valuable genetic resource for livestock, Chinese indigenous pig breeds are valuable genetic resources because of their environmental adaptability, meat quality, and reproductive performance [1,2,3,4]. Meishan pigs are particularly well known for their high litter size and have therefore been used as an important maternal genetic resource in pig breeding [5,6,7]. However, litter size is a complex quantitative trait influenced by multiple genes, parity, season, year, and other environmental factors [8,9]. Identifying genetic variants and candidate genes associated with litter size may improve the understanding and utilization of the reproductive characteristics of Meishan pigs and provide molecular information for genetic selection.

With the rapid development of modern livestock farming and commercial breeding systems, transforming the naturally occurring reproductive advantages of indigenous pig breeds into practical, selectable, and quantifiable molecular markers has become a pressing scientific issue in animal genetics and breeding. In this context, population genomics analysis based on single nucleotide polymorphism (SNP) chips—particularly genome-wide association studies (GWAS)—has emerged as a key technological approach for elucidating the genetic basis of important economic traits in livestock and poultry and identifying genes associated with high reproductive performance, and has been successfully applied in animal breeding [10,11,12]. Moreover, liquid-phase SNP chips provide a flexible platform for customized genotyping through solution-based probe hybridization and targeted sequencing [13]. Liquid-phase chips based on Genotyping by Target Sequencing (GBTS) [14] technology combine probe hybridization capture with high-throughput sequencing in a liquid-phase system, enabling efficient and accurate capture and detection of specific genomic target regions. Through bioinformatics analysis, precise genotype information for the target loci is obtained [15,16]. With high flexibility, cost-effectiveness, and high information density, liquid-phase chip technology is emerging as a powerful tool bridging genomics and molecular breeding practices. It has demonstrated considerable application potential, particularly in livestock species and breeds for which the development of conventional solid-phase SNP arrays is costly or existing commercial arrays are unavailable [17,18,19].

In this study, we utilized the “Shenxin No. 1” 60K porcine SNP liquid-phase chip, which we had developed earlier to perform genome-wide genotyping on 198 Meishan sows with complete reproductive records. Through genome-wide association analysis (GWAS), we scanned the entire genome to identify chromosomal regions and SNP loci significantly associated with litter size traits in Meishan pigs. We subsequently annotated the associated genomic regions and evaluated *AEBP2* expression and function in porcine ovarian granulosa cells. We hypothesized that genetic variants associated with litter size are located in specific genomic regions and that selected candidate genes may influence reproductive traits by affecting the biological behavior of porcine ovarian granulosa cells.

## 2. Materials and Methods

### 2.1. Collection of Samples and Phenotypic Data

This experiment selected 198 parous sows with complete breeding records from the Shanghai Jiading Meishan Pig Conservation Farm. Blood samples (5 mL) were collected via the jugular vein using disposable vacuum blood collection tubes containing EDTA-K2 anticoagulant and stored at −20 °C for future use. Phenotypic data were obtained from the 2018–2023 breeding records of the Shanghai Jiading Meishan Pig Breeding Center. The phenotypic indicator for litter size was defined as the average litter size calculated as [total number of piglets (in this study, the total number born (TNB) phenotype was defined as the sum of fully recorded litter size records across all valid parities for each parous Meishan sow, with abnormal parturition events including stillbirth-only litters and missing reproductive information excluded in advance)/number of litters (Table S1). Porcine ovarian granulosa cells used in the cellular experiments were sampled from the ovaries of Meishan pigs at a pig slaughterhouse. Healthy ovaries were isolated and placed in 37 °C saline containing 100 IU/mL of a dual antibiotic [penicillin and streptomycin (servicebio, Wuhan, China)], then transported back to the laboratory within 1 h for later use.

### 2.2. Genotyping and Data Quality Control

After extracting DNA from blood samples, whole-genome genotyping of the 198 sows was performed using the “Shenxin No. 1” 60K porcine SNP liquid-phase chip [16], which was developed in-house. Strict quality control was conducted using PLINK v 1.90 software, with the following uniform quality control standards: exclude individuals with a genotyping detection rate < 90%; loci with an SNP detection rate < 90%, a minor allele frequency (MAF) < 0.01, or a *p*-value < 10^−6^ in the Hardy–Weinberg equilibrium test were excluded [20]. Following quality control, 27,383 loci were removed, and 31,430 high-frequency SNP loci on autosomes were retained for subsequent association analysis.

### 2.3. Genome-Wide Association Analysis Based on the Average Litter Size of Meishan Pigs

A genome-wide association study (GWAS) of the average litter size in sows was conducted using the Mixed Linear Model (MLM) in the GEMMA (v 0.98.1) software. The model can be expressed as follows:

y = Xβ + Z_k_γ_k_ + ζ + e

where y is the vector of observed phenotypic values; Xβ represents fixed-effect terms (including population structure covariates and environmental factors such as year and season); Z_k_γ_k_: effect of the kth SNP marker under investigation; ζ: polygenic background effect; e: random residual effect; and K in the polygenic effect represents the marker-inferred kinship matrix [21].

The Bonferroni [22] method was used to correct the multiple comparison threshold. The effective number of tests, N, was defined as the number of SNPs. A total of 30805 SNPs used in the final GWAS analysis were obtained after GEMMA (v 0.98.1) quality control. The genome-wide significance threshold was set at 1.62 × 10^−6^ (0.05/30,805), and the potential association threshold was set at 3.25 × 10^−5^ (1/30,805). The Manhattan plot and Q-Q plot were generated using the R package qqman 0.1.9 [23]; the two thresholds are represented in the Manhattan plot by a red horizontal solid line and a blue horizontal solid line, respectively. Gene annotation was performed for the 200 kb regions upstream and downstream of the associated SNP loci (defined as the range from the first SNP locus to the last SNP locus) using the R packages biomaRt 2.62.1 [24] and GenomicRanges 1.58.0 [25].

### 2.4. Isolation, Culture, and Identification of Porcine Ovarian Granulosa Cells from Porcine Ovaries

Select healthy follicles with a diameter of 3–5 mm from the surface of the ovary, aspirate the follicular fluid using a syringe, and centrifuge at 1000× *g* for 10 min. Gently resuspend the pellet in HBSS (servicebio, Wuhan, China) containing dual antibodies to wash it, then centrifuge at 1500× *g* for 10 min. Resuspend the cells in DMEM/F12 (Gibco, Grand Island, NY, USA) medium containing 10% FBS (Gibco, Grand Island, NY, USA). After counting, seed the cells at a density of 4 × 10^5^ cells per well into a 6-well plate and culture in an incubator at 37 °C and 5% CO_2_. After 24 h, gently wash with HBSS to remove unadhered cells.

Given that FSHR is a marker protein specifically expressed by porcine ovarian granulosa cells [26], immunocytochemistry was used to assess cell purity. After fixing the cells with 4% paraformaldehyde (servicebio, Wuhan, China), permeabilizing them with 0.5% Triton X-100 (Solarbio, Beijing, China), and blocking with 20% BSA (Sigma-Aldrich, St. Louis, MO, USA), a rabbit polyclonal anti-FSHR antibody (Servicebio, Wuhan, China) was added and incubated overnight at 4 °C. The following day, FITC-labeled goat anti-rabbit IgG (Servicebio, Wuhan, China) was added and incubated at room temperature in the dark for 45 min, followed by DAPI (Servicebio, Wuhan, China) staining of the nuclei. After thorough washing, five fields of view were randomly selected under a fluorescence microscope, and the proportion of FSHR-positive cells relative to the total cell count was calculated.

### 2.5. Cell Transfection and Cell Proliferation Assays

When the cell density reached 50%, Lipofectamine™ 3000 (Invitrogen, Carlsbad, CA, USA) was used to transfect the cells with the empty vector pcDNA3.1(+) and the target plasmid pcDNA3.1(+)-*AEBP2* (Genepharma, Suzhou, China), respectively. Total protein was extracted 48 h after transfection, quantified using the BCA assay, and analyzed by Western blot. Primary antibodies included a rabbit anti-GAPDH monoclonal antibody and a mouse anti-*AEBP2* polyclonal antibody (both from Proteintech, Wuhan, China); the secondary antibody was an HRP-conjugated antibody (Proteintech, Wuhan, China). After ECL development, grayscale quantitative analysis of the protein bands was performed using ImageJ 1.54r, with GAPDH serving as the internal control to calculate relative expression levels. Cell proliferation was assessed using the CCK-8 assay. Porcine ovarian granulosa cells were seeded at 2 × 10^3^ cells per well in a 96-well plate and transfected when the cell density reached 50%. At 0 h, 24 h, and 48 h post-transfection, 10 μL of CCK-8 reagent (Dojindo, Kumamoto, Japan) and 90 μL of culture medium were added to each well; the plates were then incubated for an additional 3 h, and the absorbance (OD value) was measured at a wavelength of 450 nm using a microplate reader.

### 2.6. Statistical Analysis

All cytological experiments in this study included three independent biological replicates. Data were analyzed statistically using GraphPad Prism 9 software. An independent-samples two-way ANOVA was used to compare data between groups. Experimental data are expressed as “mean ± standard deviation (Mean ± SD),” and a *p*-value < 0.05 was considered statistically significant.

## 3. Results

### 3.1. Quality Control and PCA Analysis

**Figure 1:**
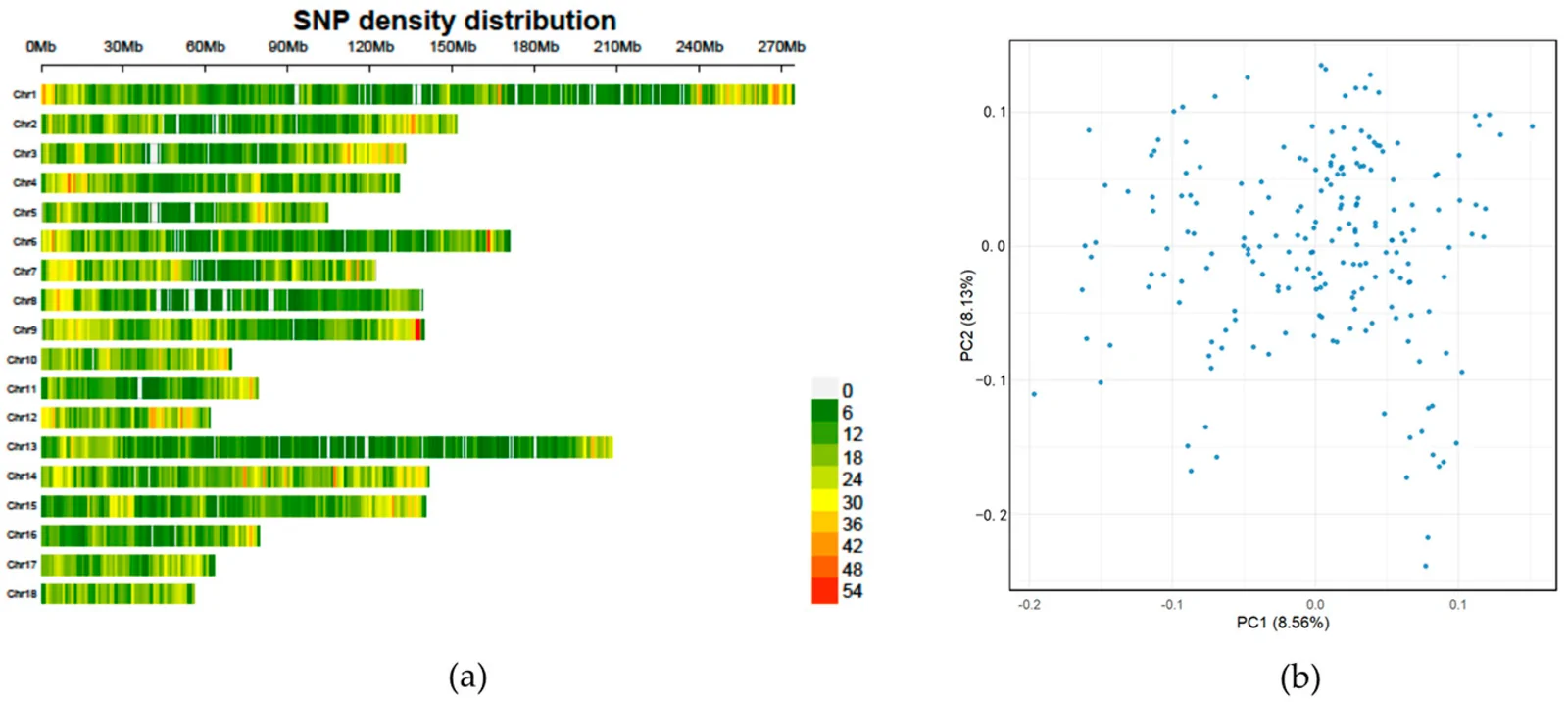
Quality control and PCA analysis of Meishan pig population data. (**a**) Distribution of SNP density on *Sus scrofa* 11.1; (**b**) PCA result graph.

Following quality control, a total of 198 individuals and 31,430 SNP loci were retained for subsequent analysis. All SNP loci were evenly distributed across the autosomes (Figure 1a). PCA results showed that no significant genetic separation was observed among the populations (Figure 1b).

### 3.2. GWAS on the Reproductive Traits of Meishan Pigs

**Figure 2:**
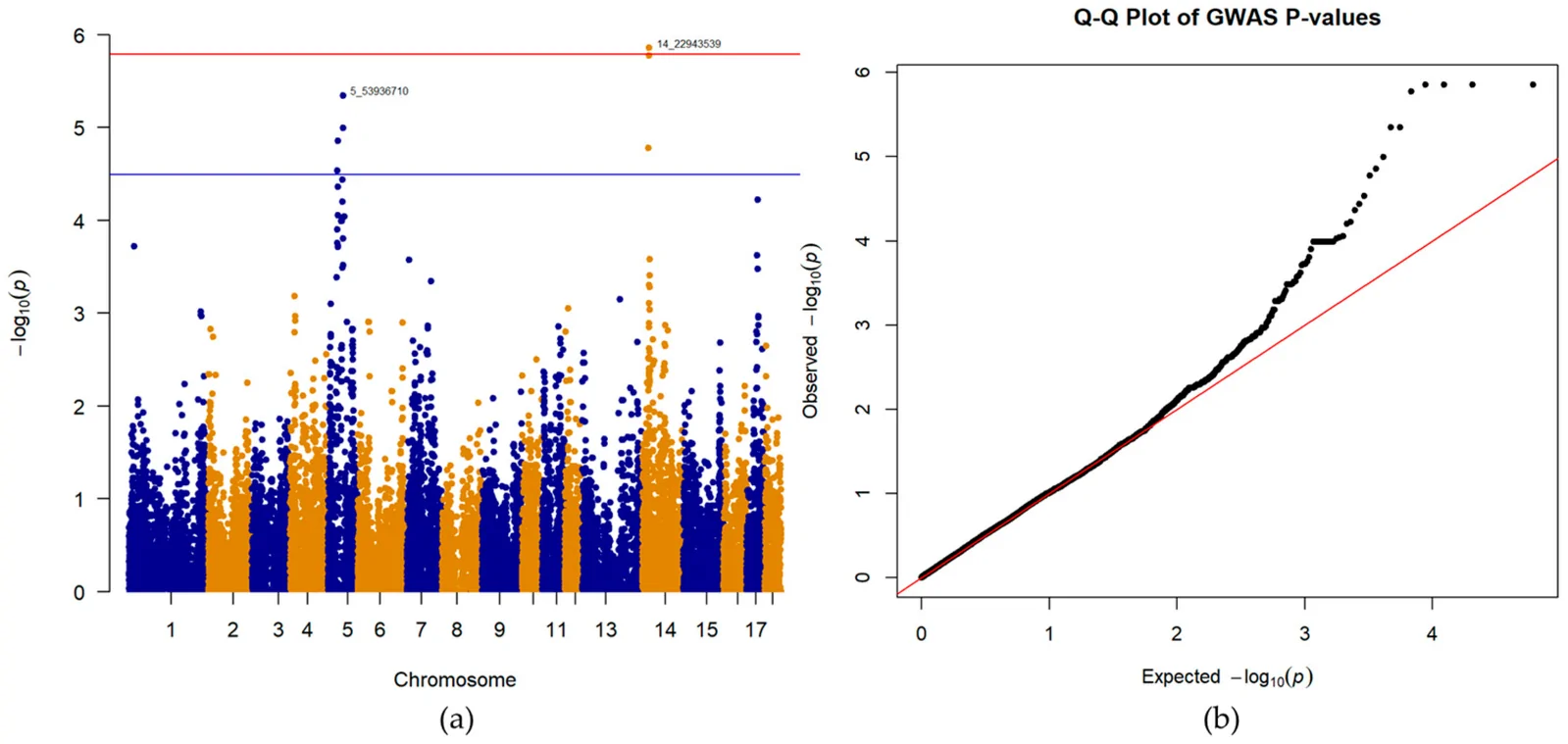
Genome-wide association analysis of litter size in Meishan pigs. (**a**) Manhattan plot of the GWAS results for the litter size trait in the Meishan pig population; (**b**) Q-Q plot.

Using a mixed linear model with the GEMMA (v 0.98.1) software to perform genome-wide SNP association analysis (Figure 2), we identified 9 SNP loci on chromosomes 5 and 14 that are potentially associated with litter size in Meishan pigs (Table 1). Gene annotation was performed on the screened SNP regions, and a total of 52 candidate genes were annotated in the significantly associated regions, including *AEBP2*, *PLEKHA5*, *MMP17*, *SFSWAP*, *POLE*, *P2RX2*, *ANKLE2*, *PGAM5*, *PXMP2*, *EP400*, *PUS1*, *GALNT9*, *SPOCK3*, *CHFR*, *GOLGA3*, and *U6*. Through the search, we found that five genes—*AEBP2*, *P2RX2*, *ANKLE2*, *PGAM5*, and *EP400*—were found to be associated with reproductive traits, primarily related to embryonic development and ovarian function.

### 3.3. Functional Validation of AEBP2 in Porcine Ovarian Granulosa Cells

#### 3.3.1. *AEBP2* Expression in Pig Tissues

**Figure 3:**
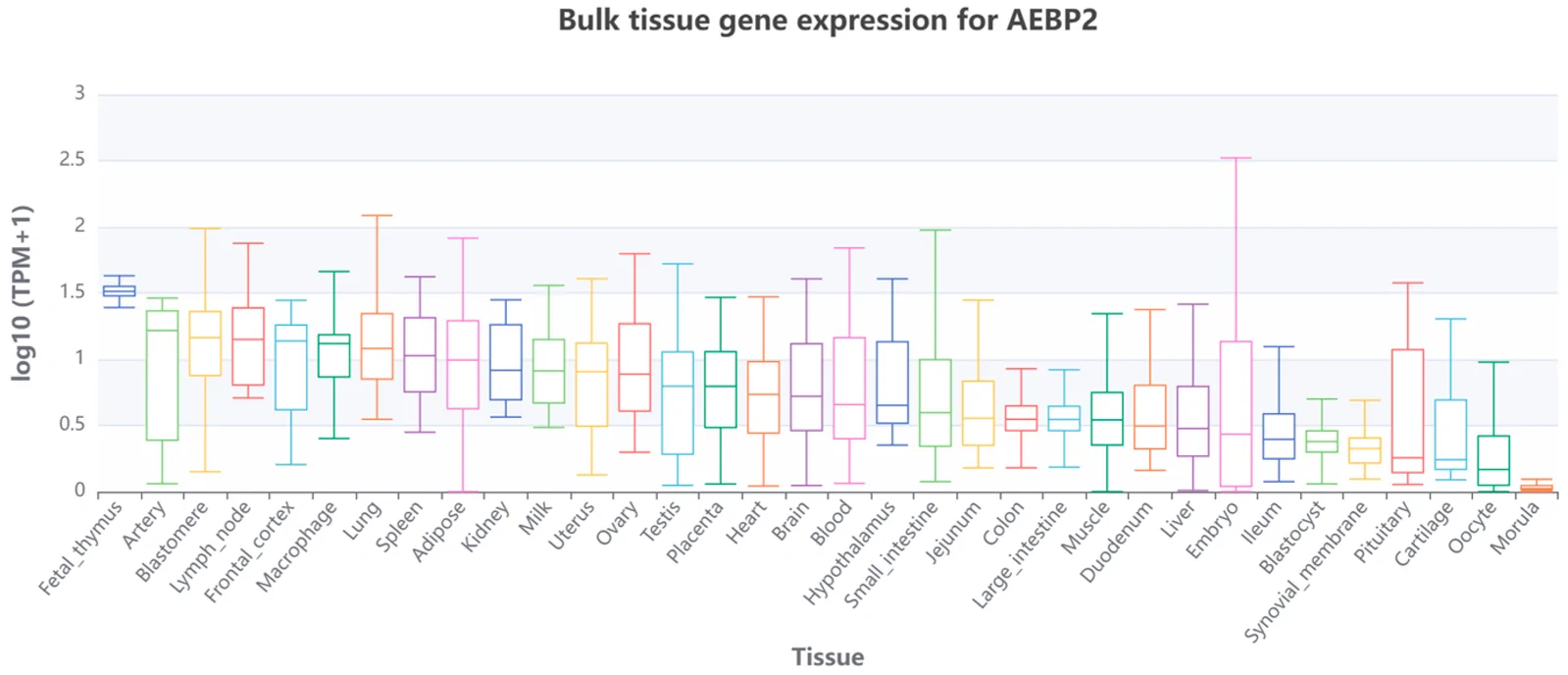
Expression profile of *AEBP2* in different tissues of pigs (search based on PigGTEx database).

Among the screened candidate genes, *AEBP2* is not only located within a region showing significant association signals, but previous studies have also demonstrated its involvement in transcriptional regulation and cellular development processes [27]. Since genes detected repeatedly across breeds exhibit higher stability and reliability, this suggests they may play a relatively conserved biological role in the formation of reproductive traits in pigs. Therefore, this study selected *AEBP2* as a key candidate gene for subsequent cellular functional validation to further clarify its regulatory role in reproduction-related cellular biological behaviors. *AEBP2* expression across pig tissues was examined using the PigGTEx database (http://piggtex.farmgtex.org) [28]. *AEBP2* expression was detected in porcine ovarian tissue and was also observed in other tissues, indicating that the gene is not ovary-specific. The tissue-expression profile is shown in Figure 3.

#### 3.3.2. Identification of Porcine Ovarian Granulosa Cells

**Figure 4:**
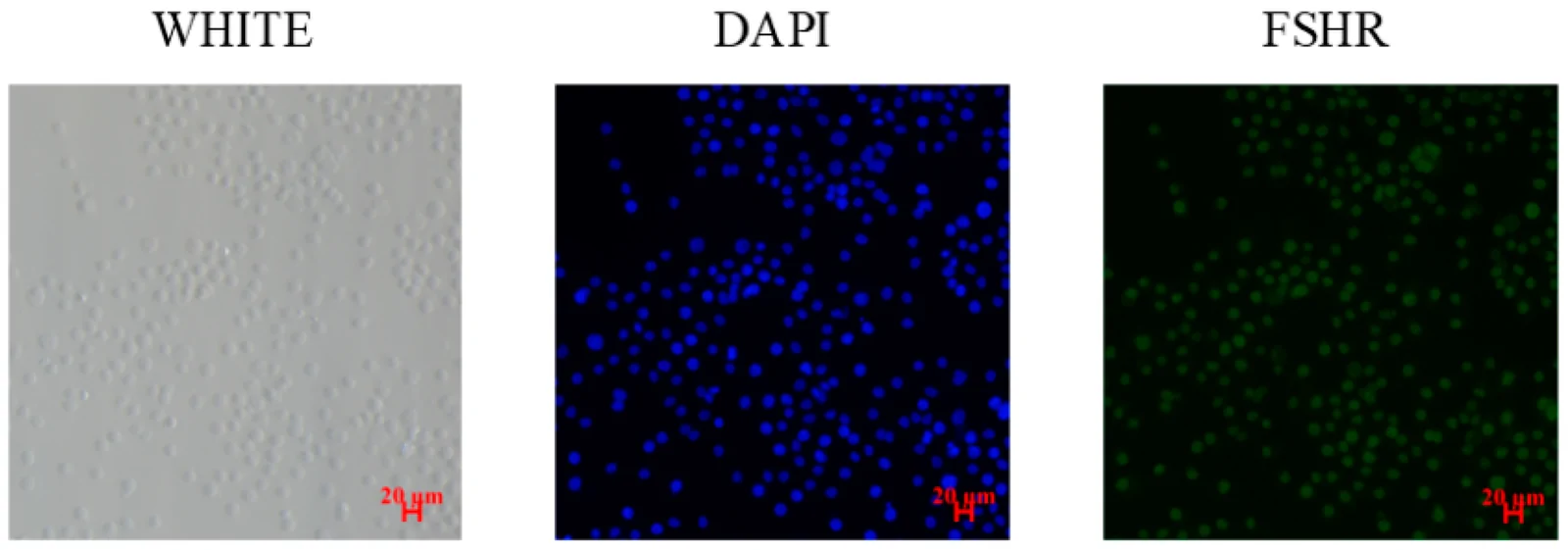
Purity identification of porcine ovarian granulosa cells.

To assess the purity of in vitro-cultured porcine ovarian granulosa cells, 4 × 10^5^ cells were seeded into a six-well plate. After 24 h of culture, cells with a confluence of 70% or higher were harvested for immunofluorescence staining. The follicle-stimulating hormone receptor (FSHR) is a marker protein expressed by granulosa cells; in this study, cells were stained with an FSHR antibody, followed by DAPI staining of the cell nuclei. The results showed that 99% of the cells were FSHR-positive (Figure 4). These findings indicate that the in vitro-cultured porcine ovarian granulosa cells exhibit high purity and are suitable for subsequent experiments.

#### 3.3.3. The Effect of *AEBP2* Overexpression on Granule Cell Proliferation

**Figure 5:**
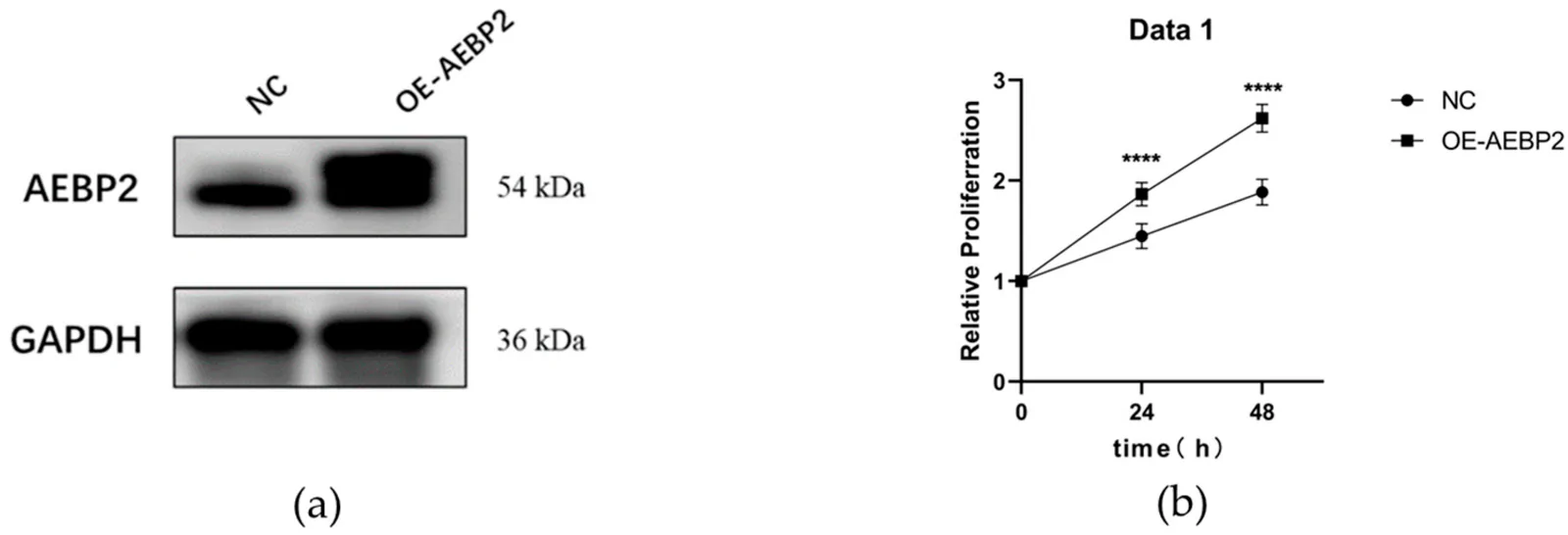
Effects of *AEBP2* on the proliferation of porcine ovarian granulosa cells. (**a**) Western blotting was used to detect the protein expression level of overexpressed *AEBP2*; (**b**) CCK-8 was used to detect the proliferation of porcine ovarian granulosa cells. Data are expressed as mean ± SEM (n ≥ 3), **** *p* < 0.0001.

Cells were transfected with either the empty pcDNA3.1(+) vector or the pcDNA3.1(+)-*AEBP2* vector, respectively. Western blot analysis confirmed that, compared with the vector control group, the AEBP2 protein expression level was elevated in the overexpression group. CCK-8 assays conducted at 0, 24, and 48 h showed that the proliferative capacity of cells in the overexpression group was significantly higher than that of the vector control group. Data are presented as [mean ± standard deviation/mean ± standard error] and are derived from three independent biological replicates, each comprising five technical replicates. Exact *p*-values are shown in Figure 5.

## 4. Discussion

In recent years, population genomics analysis based on SNP microarrays has become a key method for elucidating the genetic basis of important economic traits in livestock and poultry. For example, Wang et al. [29] genotyped 2655 Large White pigs using the GeneSeek Porcine 50K SNP chip and combined genotype imputation with genome-wide association analysis to investigate the total number born, number of stillborn piglets, and gestation length. Their analysis identified several candidate genes associated with reproductive traits, including *NOTCH2*, *KLF3*, *PLXDC2*, and *ACVR2A* for total number born, demonstrating the value of SNP-chip data and imputation strategies for dissecting the genetic architecture of reproductive performance. Ding et al. [30] conducted a large-scale GWAS in 6550 Duroc pigs genotyped with the GeneSeek Porcine 50K SNP chip and identified 80 SNPs and six quantitative trait loci associated with backfat thickness. In this study, candidate genomic regions and genes associated with average litter size were identified in a Meishan sow population, and *AEBP2* overexpression was associated with increased proliferation of porcine ovarian granulosa cells in vitro.

In the GWAS analysis, annotation of the associated genomic regions identified 52 positional candidate genes. Five of these genes—*AEBP2*, *P2RX2*, *ANKLE2*, *PGAM5*, and *EP400*—were supported by previous literature as potentially related to reproductive traits in different species or biological models. *P2RX2* mediates the direct stimulation of Kiss1 neurons in the AVPV region by purinergic neurons; its knockdown inhibits the luteinizing hormone (LH) surge, disrupts the estrous cycle, and suppresses ovulation [31]; Studies in Drosophila have demonstrated that maternally contributed *ANKLE2* is essential for early embryonic development, with its depletion causing first-mitotic-cycle arrest in most embryos and failure to hatch [32]; High expression of *PGAM5* may help maintain and regulate mitochondrial dynamics and metabolic reprogramming in aging ovarian granulosa cells, the ovaries of aged female mice, and elderly patients [33]; In mice, maternal *EP400* is required for oocyte quality, zygotic genome activation, and early embryonic development; depletion of *EP400* in oocytes impairs fertilization and causes developmental arrest at the 2–4-cell stage [34]; *AEBP2* is an evolutionarily conserved zinc-finger protein associated with PRC2 and has been proposed to contribute to its targeting to chromatin [35]. Functional studies in mouse embryos and embryonic stem cells further indicate that *AEBP2* regulates PRC2 subcomplex composition and PRC2-mediated H3K27 methylation, with implications for embryonic development [27].

Previously, Cai et al. [36] also pointed out through a meta-analysis of Jinhua pigs and Shengxian flower pigs that *AEBP2* and other genes may be candidate genes influencing litter size. Furthermore, a search of the PigGTEx database revealed that the *AEBP2* gene is expressed in porcine ovarian tissue. These repeated findings across breeds and the tissue expression profile suggest that *AEBP2* and its surrounding genetic variations may play a relatively conserved biological role in porcine reproductive traits. In this study, we established a porcine ovarian granulosa cell model overexpressing *AEBP2* and conducted CCK-8 experiments, which demonstrated that *AEBP2* promotes granulosa cell proliferation. Porcine ovarian granulosa cells are the core somatic cells involved in follicular development; they directly participate in oocyte nutrition, steroid hormone synthesis, and follicular selection. Their proliferation and apoptosis dynamically determine dominant follicle formation, follicular atresia, and the number of ovulated oocytes [37,38]. Therefore, validating the effect of *AEBP2* on cell proliferation in porcine ovarian granulosa cells can effectively reflect its potential role in reproductive regulation.

The experimental results support the hypothesis that *AEBP2* is involved in regulating reproductive traits. Based on GWAS mapping and cellular experiments, it is hypothesized that *AEBP2* influences litter size in sows by promoting granulosa cell proliferation, improving follicular development, and exerting epigenetic regulation. However, given the limited overall sample size and the fact that litter size may be influenced by multiple factors, the effects of *AEBP2* on litter size in pigs and its specific mechanisms of action require further elucidation.

It is worth noting that this study has several limitations. First, the GWAS included 198 Meishan sows, which is smaller than the sample sizes used in several large-scale pig GWASs. The relatively limited sample size may reduce statistical power for detecting variants with small effects and may lead to less stable effect estimates. Second, the use of average litter size across multiple parities may reduce information about parity-specific genetic and environmental effects. Third, the SNP associations identified here should be interpreted as statistical associations rather than evidence of causality. In particular, suggestive associations should be distinguished from genome-wide significant associations. Independent populations, larger sample sizes, and in vivo functional studies are needed to validate the identified loci and candidate genes.

## 5. Conclusions

This study utilized an SNP liquid-phase microarray to investigate the genetic basis of litter size in 198 Meishan sows. After completing quality control and association analysis, a total of 9 SNP signals were found to be associated with average litter size at the genome-wide significant or suggestive association levels. After annotating the associated regions, 52 candidate genes were identified, including *AEBP2*, *P2RX2*, *ANKLE2*, *PGAM5*, and *EP400*. Based on PigGTEx data, *AEBP2* expression was detected in porcine ovarian tissue. In vitro experiments showed that *AEBP2* overexpression was associated with increased proliferation of porcine ovarian granulosa cells. These results support the selection of *AEBP2* as a candidate gene for further research on reproductive traits in Meishan pigs. However, the current data are insufficient to establish a causal relationship between *AEBP2* and litter size in pigs; further validation in independent populations, along with additional in vivo experiments and studies on molecular mechanisms, is still required.

## Figures and Tables

**Table 1:** SNPs loci highly correlated with litter size.

Chromosomes	Position/bp	Base Mutation	*p*-Value
5	53936710	G/T	4.51 × 10^−06^
	53939587	G/T	4.51 × 10^−06^
	54059989	C/T	1.01 × 10^−05^
14	21903447	A/G	1.67 × 10^−05^
	22943539	A/G	1.39 × 10^−06^
	22944573	A/G	1.67 × 10^−06^
	22946422	A/G	1.39 × 10^−06^
	23005461	A/T	1.39 × 10^−06^
	23005470	G/A	1.39 × 10^−06^

## Data Availability

The original contributions presented in this study are included in the article. Further inquiries can be directed to the corresponding authors.

## Supplementary Materials

The following supporting information can be downloaded at: https://www.mdpi.com/article/10.3390/ani16203198/s1, Table S1: Phenotype data.
